# Patterns of health care use and out-of-pocket payments among general population and social security beneficiaries in Myanmar

**DOI:** 10.1186/s12913-019-4071-8

**Published:** 2019-04-27

**Authors:** Chaw-Yin Myint, Milena Pavlova, Wim Groot

**Affiliations:** 1 Department of Health Services Research, CAPHRI School for Public Health and Primary Care, Maastricht University Medical Center, Faculty of Health, Medicine and Life Sciences, P.O. Box 616, 6200 MD Maastricht, The Netherlands; 2 Water, Research and Training Center (WRTC), Yangon, Myanmar; 3 Top Institute Evidence-Based Education Research (TIER), Maastricht University, Maastricht, The Netherlands

**Keywords:** Social security scheme, Myanmar, Out of pocket payment, Type of health care facility, Utilization of health care

## Abstract

**Background:**

As a consequence of the low government expenditure and limited access to health insurance offered by the Social Security Scheme (SSS), out-of-pocket payments (OOPPs) have become the main source of payment for health care in Myanmar. This study aims to provide evidence on the patterns of health care use and OOPPs by the general population and SSS beneficiaries in Myanmar.

**Method:**

Face-to-face interviews were conducted among two samples drawn independently of each other. The first sample, the general population sample of persons not insured by SSS, was drawn from the general population in the Yangon Region. The second sample, the SSS sample, was drawn from those possessing SSS insurance. The data were analyzed per sample. Mann-Whitney U tests were applied to compare ordinal variables and independent sample t-tests were applied to compare continuous variables between the two samples. Two-step cluster analysis was applied to identify clusters of respondents with similar patterns of health care use and OOPPs. After the clustering procedure, we used regression analysis to examine the association between socio-demographic characteristics and cluster membership (patterns of health care use and OOPPs) for the two samples separately.

**Results:**

Only 23% of those who belonged to the SSS sample and sought health care during the past 12 months, report receiving health care from a SSS clinic during the last episode of illness. Close distance is the main reason for choosing a specific health facility in both samples. OOPPs for health care and pharmaceuticals, used during the last episode of illness are significantly higher in the general population sample. The regression analysis shows that the pattern of health care use is significantly associated with household income. In addition, respondents in the general population sample with a higher income pay higher amounts for their last health care used and were significantly more likely to have to borrow money or sell assets as a coping strategy to cover the payments.

**Conclusion:**

Significantly higher OOPPs in the general population sample highlight the need of financial protection among this group. Myanmar needs to extend social protection for both coverage breadths and coverage depth.

**Electronic supplementary material:**

The online version of this article (10.1186/s12913-019-4071-8) contains supplementary material, which is available to authorized users.

## Background

The government of Myanmar, one of the poorest countries in the world with a high burden of both communicable and non-communicable diseases, has committed itself to achieving universal health coverage (UHC) by 2030 [15]. Currently, the country has a pluralistic and fragmented mix of public and private health care systems. The sources of health care financing consist of government funding, out-of-pocket payments (OOPPs) by households, funding from the Social Security Scheme (SSS), community contributions and external aid. The government health expenditure in the 2012–2013 fiscal years was 0.76% of GDP or 3.14% of the general government expenditure (GGE). Nevertheless, these rates were substantially higher than those in 2011–2012 (0.2% of GDP or 1.05% of GGE respectively). Overall, government health expenditure is low and covers only about a quarter of the total health expenditure [24]. The result is a high share of OOPPs (50.7% of the total health expenditure). High OOPPs can lead to catastrophic expenditure especially among the poor [22, 25, 26, 27].

Currently, there is only one health insurance scheme in Myanmar, the Social Security Service SSS. The Social Security Board (SSB) was created in 1956 after the adoption of the Social Security Act (1954), which stated that factories, workshops and enterprises with more than five employees whether state owned, private, foreign or joint ventures, must provide their employees with social security coverage under the SSS [21].

SSS is available in all regions except Chin state where such services are not accessible. However, only 706,750 employees out of a total 21.8 million employees in the country (i.e. only 3.2%) are covered under the SSS [12, 21]. Because of governance shortcomings, a significant number of employees are outside any social or health insurance system especially employees in the informal sector. Consequently, the SSB has prepared a Social Security Law (2012) to increase insurance coverage through compulsory SSS contributions from the formal sector as well as voluntary SSS contributions from the informal sector and the community [11, 18, 21].

Employees insured under the SSS, are provided with free medical treatment (based on a benefit package), cash benefits for sickness absence and maternity leave and occupational injury benefits. The new Social Security Law (2012) extended the existing benefit package; with higher cash benefits for sickness, maternity and work injury included; access to medical facilities outside the SSB-owned facilities; and eligibility of smaller enterprises and voluntary registration of family members of employees, students and informal workers. The benefit package included medical treatment (out-patient, in-patient, medicine, laboratory, transportation in case of referral outside urban areas) for a maximum of 26 weeks. Free access to all SSB facilities except for retired workers who have to pay co-payments equal to 50% of the cost of treatment. Reimbursement by SSS is based on fixed rates in case of a referral to public facilities. Medical care of newborns is covered up to 1 year of age.

Aside from the low health insurance coverage, a recent evaluation of the SSS showed other major weaknesses, such as the low cash benefits for the SSS members as well as the contradicting objectives because SSS has to play multiple roles in the financing of health care, such as pooling of risks and purchasing and providing health care services. Furthermore, the network of health care providers under the SSS, is limited and overall the beneficiaries perceive the quality of SSS services as low. The IT system of the SSS is not well-established. Financial projection tools are not yet available for the SSS. All these factors contribute to the SSS low enrolment rate [21]. As a consequence of the low government expenditure and the limited SSS coverage, OOPPs have become the main source of payment for health care in Myanmar. Studies show that nearly 41% of the households in Myanmar experience catastrophic expenditures when using health care [7, 8]. One study conducted in Mandalay City found that the incidence of households’ catastrophic health care payment is 8, 4 and 1.3% for the three catastrophic thresholds of 10, 20 and 30% of household income, respectively [3]. Evidence shows that family income, education, age of household’s head, age and gender of the ill person, and patients’ perceived quality of care play a role in choosing the type of health care services [2, 13, 14].

Although there was a recent evaluation of the SSS [21] and there are several studies on the factors influencing the utilization of health care [5, 17, 23], there is no evidence on how the SSS members use and pay for health care, and to what extent they make use of insurance coverage. This study aims to identify patterns of health care utilization and OOPPs among SSS members and those among the general population living in the Yangon Division with no SSS insurance. We also analyze the association between socio-demographic characteristics and patterns of health care utilization and payments. The results throw light on the implications of the policies to move towards UHC in Myanmar. Policy-makers and researchers in other countries where the extension of health coverage is on the policy agenda could also benefit from getting informed about the Myanmar case.

## Methods

We used data from a survey carried out through face-to-face interviews in June–August 2015 among two population groups, namely among the general adult population (18+) in the Yangon Region who are not SSS members, and among persons insured under the SSS. The survey collected data on health care use and payments, as well as perceptions and preferences for health insurance. In this paper, we use the survey data to explore the patterns of health care use and OOPPs among those with and without SSS coverage.

### Sample size calculation

The sample size for each group is calculated by using the simple formula:

$$ \mathrm{n}=\frac{Z_{\raisebox{1ex}{$\alpha $}\!\left/ \!\raisebox{-1ex}{$2$}\right.}^2\mathrm{P}\left(1-\mathrm{P}\right)}{{\mathrm{d}}^2}, $$

Where *n* is the sample size, *Z*_*α/2*_ in the z statistics for a level of significance *α* (we used *α* = 0.05, and thus, *Z*_*0.05/2*_ = 1.96), *P* is the proportion of target population accessing essential health services (we used the proportion of child births with skilled birth attendance, which is 0.7 in Myanmar), and *d* is precision (acceptable error), which we set to be 0.05. Thus, the required sample size was ca. Three hundred twenty respondents. We applied the same calculation procedure for both groups.

### Sampling procedure

Each population group was sampled separately. Thus, we carried out two separate sampling procedures. The first sample, the general population sample, was drawn from the general population in the Yangon Region with no SSS coverage. The second sample, the SSS sample, was drawn from the group of those with SSS insurance. Below a brief summary of the sampling procedures is given.

#### General population

The target population consisted of adults living in the Yangon Region, Myanmar and not possessing SSS insurance. For the general population sample, a multistage group sampling procedure was performed, which included the selection of townships, followed by the selection of wards within the townships, and finally, the selection of households within the selected wards. Thus, the sampling procedure consisted of three stages. In the first stage, four townships in the Yangon Division were selected with different characteristics: two townships in urban areas, Bahan and Ahlone, and two townships in suburban areas, North Dagon and Hlegu, to capture the responses of persons living in different settings. In the second stage, four wards inside each township were selected by the local authorities according to feasibility. In the third stage, households inside the wards were randomly selected in the following way: from a bowl, a number from one to six could be picked. This number determined how many houses would be skipped by the surveyors to select the next participant. The sampling method excluded all those who did not have an address, e.g. the homeless people. Furthermore, only adults over the age of eighteen were included. We skipped respondents if the household had SSS insurance.

The head of the household or the main decision maker in each selected household was asked to participate in the face-to-face interview using a standardized questionnaire. If an individual was not able or willing to participate, a replacement was identified following the same sampling procedure, to avoid a waste of resources. The procedure ended when 320 interviews were carried out.

#### SSS population

The target population consisted of employees enrolled in the SSS. For the SSS sample, a multistage sampling procedure was performed, which included the selection of area office, which was followed by the selection of the type of employer (government or non-government), and finally the selection of respondents. Thus, the sampling procedure consisted of three stages. In the first stage, according to a generated random number, 4 area offices were randomly chosen from the 77 area offices covered by the SSS in the country. These were Shwe Pyi Thar, Office 5, Kyaut Se, and Bago, In the second stage, employer organizations were selected as follows: 3 government owned and 3 non-government owned organizations in the area offices Shwe Pyi Thar and Office 5, and 2 government owned and 2 non-government owned organizations in the areas offices Kyaut Se and Bago. The criterion for selecting these organizations was that the organization consisted of at least 10 employees and could be easily approached. In the third stage, we randomly selected 7–9 respondents from the list of employees in each government owned organization and 24–26 respondents from the list of employees in each non-government owned organization. The selected respondents were interviewed using the same structured questionnaire as for the general population. The number of organizations and participants was calculated based on the ratio of the distribution of employee in government owned / non-government owned organizations. The procedure ended when 320 interviews were carried out.

### The questionnaire

The questionnaire was developed in English and then translated into Burmese. Backward translation was done in English to check the quality and to correct the Burmese version. A pilot study was done with a sample of 30 participants to pre-test the face validity of the questionnaire. The questionnaire was adjusted accordingly. The questions were identical for both samples. The questionnaire consisted of five parts related to respondents’ socio-demographic characteristics; past health care utilization and payments; knowledge, attitudes and practice of health insurance; willingness and ability to pay for health care services; and preferences to pay for health care services.

In this paper, we only analyze the data on past health care use and OOPPs. Related to this, the questionnaire included questions about the last episode of illness and the type of health care used, as well as questions about the reasons of health care utilization at a specific health center/hospital (e.g., distance, quality, cost, recommended, etc.), method of payment (e.g., OOPPs, SSS resources, community-based health insurance (CBHI), etc.), the amount of payment and coping strategies such as whether respondents borrowed money or sold assets to pay for health care. All these questions referred to the respondents’ last illness within the preceding 12 months.

The English wording of the questions used in this paper such as questions on socio-demographic factors and health seeking behaviors during the past 12 months are provided in Additional file 1.

### Data collection

The interviewers received one-day training on the fieldwork standards and the specificities of the questionnaire including the basics concepts of health insurance. There were face-to-face interviews with the participants performed by the Burmese speaking interviewers to fill out the questionnaire. The respondents received an explanation of the survey objectives and a confirmation about the confidentiality of the survey data. The participants were asked for an informed consent prior to the interviews.

### Statistical analysis

We started the analysis by comparing the socio-demographic characteristics of the two samples, the general population sample and the SSS sample. This was done to establish the differences between the samples, some of which were expected, such as differences in age and gender. This is because SSS coverage can only be obtained by a specific group of working-age individuals, as explained at the outset of this paper. Mann-Whitney U test was applied to compare ordinal variables and independent sample t-test was applied to compare continuous variables between two samples.

The rest of the data from the two samples, were also analyzed separately by descriptive statistics for each variable for each sample, and comparing these results between the two samples. Again, Mann-Whitney U test was applied to compare ordinal variables and independent sample t-test was applied to compare continuous variables between the two samples.

Next, we used two-step cluster analysis (Software package SPSS 27) to cluster the respondents based on their individual responses (separately for each sample). The algorithm employed by the two-step clustering procedure is suitable for large datasets containing categorical and continuous variables data like our dataset. It also calculates the optimal number of clusters by comparing the values of a model-choice criterion across different clustering solutions. Thus, the Schwarz’s Bayesian Information Criterion (BIC) method was applied in the two-step clustering procedure to determine the clusters. We did not predefine the number of clusters. BIC is a criterion for model selection based on comparing the values of the likelihood functions. The model with the lowest BIC is preferred.

Four cluster analyses were carried out per sample. Cluster analysis I was based on a combination of all variables related to the last health care utilization in the past 12 months and payments reported by the respondents. These variables were then divided into three groups: [1] variables indicating the type of services used during the last episode of illness, and reasons for the choice of these services, [2] variables indicating the payment type, and [10] variables indicating the burden of OOPPs, such as size of OOPPs and coping strategies. Thus, cluster analysis II was based on the type of services and reasons of using these services; cluster analysis III was based on the type of payment for health care services used, e.g. OOPPs, SSS resources, CBHI contributions, etc.; and cluster analysis IV was based on the amount of OPPPs and coping strategies applied to deal with the high OOPPs. The questions and response categories in the four cluster analyses, are described in Table 1.

**Table 1:** English wording of the questions used in the survey to obtain data for the cluster analysis

Cluster analysis I	Combination of all cluster variables from cluster analysis II, III and IV.
Cluster analysis II (Type of health services used for last illness during the past 12 months and reason of using these services)	Which of the following service types can best describe your last use of health care services? (select only one service type related to the very last use of health care services)
	• Visit to nearby health center
	• Visit to general practitioner
	• Visit to outpatient medical specialist at public hospital
	• Visit to outpatient medical specialist at private hospital
	• Hospitalization (incl. One day hospitalization)
	• Traditional healer
	• Other, please specify: ……………….
	Why did you choose this kind of health service? (yes/no)
	• It was the closest facility
	• I had to pay less than in other facilities
	• I had to wait less than in other facilities
	• It provided the best quality services
	• It was recommended to me
	• I was brought there
	• Other, please specify: ……………….
Cluster analysis III (type of payment for health care services)	Which kinds of methods were used to cover all costs related to your last use of health care services? (multiple answers possible) (yes/no)
	• Social security scheme (SSS)
	• Community based health insurance (CBHI)
	• Out-of-pocket payments (OPPs)
	• Other, please specify: ……………………
Cluster analysis IV (amount of out-of-pocket payments and coping strategies)	How much did you spend in total for your last use of health care services?
	How much of this was for pharmaceuticals (medicines)?
	Did you have to borrow money to cover the above expenses for your last use of health care services and pharmaceuticals? (yes/no)
	Did you have to sell assets to cover the above expenses for your last use of health care services? (yes/no)

The two-step cluster analysis procedure that we applied, specified the clustering quality based on the Silhouette Index (SI). The SI indicates how well each subject/object lies within its cluster, and thus, it validates the clustering outcomes. SI ranges from − 1 to 1. SI greater or equal to 0.5 indicates good clustering quality.

After the formation of the clusters in each cluster analysis, we applied binary logistic regression when the dependent cluster-membership variable was binary. This was done for cluster analysis I for both sample groups, as well as for cluster analysis II for the SSS sample, cluster analysis III and IV for both sample groups. The multinomial logistic regression was used for cluster analysis II of the general population sample where the dependent cluster-membership variable was nominal with more than two levels (more than two clusters). The regression analysis was done to examine the association between socio-demographic characteristics and cluster memberships generated in the cluster analysis (for the SSS sample and the general population sample separately). This regression analysis was done to identify if a cluster of respondents with a specific pattern of health care use and/or specific pattern of OOPPs, belong to similar socio/demographic groups. Socio-demographic characteristics of health care use and OOPPs identified in previous studies (Aung et al. Janurary 2016; [13, 14]), were taken into account. These characteristics included age, gender, education of the respondent, occupation, civil status, current health status, number of adult persons in the household, number of children in the household, logarithm of the monthly net household income, and household expenditure.

### Ethical clearance

Ethical clearance was obtained from Ethical Committee of Lower Myanmar Research Department.

## Results

### Socio-demographic characteristics

The mean age of the respondents in the general population sample is 50 years and is higher than that of the SSS sample which is 33 years. The difference in mean age between the two samples is significant at *p* < .001.The general population sample includes 187 (58.4%) women and 133 (41.6%) men while the SSS sample includes 213 (66.6%) women and 107 (33.4%) men respectively. A high rate of unemployed and informal workers is found in the general population sample (33.1 and 30.6% respectively). In the SSS sample, the proportion of employees in public (government owned organizations) and private (non-government owned organizations) reflects the nationwide proportion which is 25:75. The distribution of respondents over education levels is fairly similar, but the share of respondents with high school and higher education is higher in the SSS sample. About 70% of the respondents in the general population sample are married while 50% of the respondents of the SSS sample are single. Around 50% of the respondents in both samples rate their current health status as good. The average number of adults and children in the household is 4 and 1 respectively in the two samples. The average household income in the general population sample is significantly higher, i.e., 434,698 MMK, than that in the SSS sample, i.e., 335,073 MMK, (*p* < .001). More than 30% of the respondents in the SSS sample can save some amount of their income during a month while less than 20% of the general population sample can save some money. The socio-demographic characteristics of the two samples are summarized in Table 2.

**Table 2:** Socio-demographic characteristics

			General population sample	SSS population sample	Significance of the differences between the samples
Age	Years		*N* = 320	*N* = 320	
		Median	50	31	.001^b^
		Mean	50	33	
		SD	15	11	
Gender			*N* = 320	*N* = 320	
	Male	*N* (%)	133 (41.6%)	107 (33.4%)	.034^a^
	Female	*N* (%)	187 (58.4%)	213 (66.6%)	
Occupation			*N* = 320	*N* = 320	
	Public	*N* (%)	12 (3.8%)	80 (25%)	
	Private	*N* (%)	24 (7.5%)	240 (75%)	
	Self-employed	*N* (%)	98 (30.5%)	–	
	Family Business	*N* (%)	37 (11.6%)	–	
	Pension	*N* (%)	29 (9.1%)	–	
	Students	*N* (%)	4 (1.3%)	–	
	Unemployed	*N* (%)	106 (33.1%)	–	
	Other	*N* (%)	10 (3.1%)	–	
Education			*N* = 320	*N* = 320	
	Illiterate	*N* (%)	5 (1.6%)	2 (0.6%)	.445^b^
	Primary School	*N* (%)	36 (11.3%)	18 (5.6%)	
	Middle School	*N* (%)	62 (19.4%)	61 (19.1%)	
	High School	*N* (%)	114 (35.6%)	109 (34%)	
	Graduate and Higher degree	*N* (%)	98 (30.5%)	124 (38.8%)	
	Other	*N* (%)	5 (1.6%)	6 (1.9%)	
Civil Status			*N* = 320	*N* = 320	
	Single	*N* (%)	48 (15%)	166 (51.8%)	
	Married	*N* (%)	230 (71.9%)	144 (45.1%)	
	Living with a partner without marriage	*N* (%)	–	3 (0.9%)	
	Separated	*N* (%)	3 (0.9%)	3 (0.9%)	
	Divorced	*N* (%)	4 (1.3%)	–	
	Widow	*N* (%)	34 (10.6%)	4 (1.3%)	
	No answer	*N* (%)	1 (0.3%)	–	
Self-reported health status			*N* = 320	*N* = 320	
	Very poor	*N* (%)	5 (1.6%)	2 (0.6%)	.767^b^
	Poor	*N* (%)	32 (10.0%)	30 (9.4%)	
	Moderate	*N* (%)	105 (32.8%)	121 (37.8%)	
	Good	*N* (%)	166 (51.8%)	149 (46.6%)	
	Very good	*N* (%)	12 (3.8%)	18 (5.6%)	
Adult persons in the households	Number of person		*N* = 319	*N* = 320	
		Median	3	3	.300^b^
		Mean	4	4	
		SD	2	2	
Under 18 years in the households	Number of person		*N* = 249	*N* = 314	
		Median	1	1	.608^b^
		Mean	1	1	
		SD	1	1	
Average household income per month	Amount (MMK)^c^		*N* = 314	*N* = 320	
		Median	300,000	300,000	.001^b^
		Mean	434,698	335,073	
		SD	477,764	201,507	
Level of income after household expenditure			*N* = 320	*N* = 320	
	Savings	*N* (%)	12 (3.8%)	9 (2.8%)	.189^b^
	Save a little	*N* (%)	48 (15%)	101 (31.5%)	
	Meet the expenses	N (%)	219 (68.4%)	173 (54.1%)	
	Not sufficient/need to use saving	*N* (%)	10 (3.1%)	6 (1.9%)	
	Not sufficient/need to borrow	*N* (%)	20 (6.3%)	31 (9.7%)	
	No answer	*N* (%)	11 (3.4%)	–	

^a^Mann-Whitney U Test; ^b^ Independent samples t-test; ^c^ Exchange rate 1000 MMK = 0.81 USD (2015)

### Health care utilization during the last episode of illness

Health care use during the last episode of illness in the past 12 months among the two samples is described in Table 3. There is a statistically significant difference between the two samples in terms of the share of respondents who reported illness during the past 12 months (38.4% of the general population sample and 55.3% of the SSS sample). Among these, 87% of the general population sample and 90.4% of the SSS sample used health care, and these rates are fairly similar showing no significant difference between the samples (*p* = 0.32). Various types of health care facilities are used during the last episode of illness. For example, 23% of the SSS respondents who used health care, report receiving health care from a SSS clinic. In the general population sample, for example, the use of outpatient care at a private clinic is reported by 31.8% of the respondents who used health care during their last episode of illness. Close distance is the main reason for choosing a specific health facility in both samples. However, among the general population sample, choosing a facility with the best perceived service quality is reported approximately two times more frequently than among the SSS sample. This difference is also statically significant (*p* < 0.01). The mean distances between the health facility used and the respondent’s home does not differ much between the two samples, i.e. 28 min on average for the general population sample, and 34 min on average for the SSS sample. The mean waiting time is significantly longer (*p* < 0.01) for the general population sample (46 min on average) than for the SSS sample (29 min on average). Also, 70.2% of those in the general population sample who sought health care, and 61.7% of the corresponding group in the SSS sample rate the quality of health care they received as good.

**Table 3:** Health care utilization and health expenditure

Variables			General Sample *N* (%)	SSS Sample *N* (%)	Significance of the differences between the samples
Any symptoms of illness during the past 12 months			*N* = 320	*N* = 320	
	Yes = 1		123 (38.4%)	177 (55.3%)	0.000 ^a^
	No = 0		197 (61.6%)	143 (44.7%)	
Seek/receive health care services during the past 12 months (physician visit or hospitalization)			*N* = 123	*N* = 177	
	Yes = 1		107 (87.0%)	160 (90.4%)	0.322 ^a^
	No = 0		17 (13%)	17 (9.6%)	
Types of services received (excluding respondents who did not seek/receive health services during the past 12 months)			*N* = 107	*N* = 160	
	Nearby health center		21 (19.6%)	26 (16.3%)	
	General Practitioner		36 (33.6%)	72 (45.0%)	
	Outpatient care (public)		8 (7.5%)	8 (5.0%)	
	Outpatient care (private)		34 (31.8%)	7 (4.4%)	
	Hospital		5 (4.7%)	7 (4.4%)	
	Traditional healer		1 (0.9%)	–	
	Abroad		2 (1.9%)	–	
	Clinic in industry		–	1 (0.6%)	
	NGO owned hospital		–	1(0.6%)	
	Go to Health Assistant		–	1 (0.6%)	
	SSS clinic			37 (23.1%)	
Reasons to choose health facility (excluding respondents who received health care from traditional healer and others)			*N* = 104	*N* = 120	
	Close facility	Yes = 1	55 (52.9%)	70 (58.3%)	0.414 ^a^
		No = 0	49 (47.1%)	50 (41.7%)	
	Low cost	Yes = 1	16 (15.4%)	11 (9.2%)	
		No = 0	88 (84.6%)	109 (90.8%)	0.155 ^a^
	Short waiting time	Yes = 1	5 (4.8%)	1 (0.8%)	0.067 ^a^
		No = 0	99 (95.2%)	119 (99.2%)	
	Best quality	Yes = 1	29 (27.9%)	15 (12.5%)	0.004 ^a^
		No = 0	75 (72.1%)	105 (87.5%)	
	Recommended	Yes = 1	9 (8.7%)	13 (10.8%)	0.585 ^a^
		No = 0	95 (91.3%)	107 (89.2%)	
	Brought by someone	Yes = 1	6 (5.8%)	7 (5.8%)	0.984 ^a^
		No = 0	98 (94.2%)	113 (94.2%)	
	Others	Yes = 1	8 (7.7%)	16 (13.3%)	0.174 ^a^
		No = 0	96 (92.3%)	104 (86.7%)	
Distance between health facility and resident (minutes)			*N* = 104	*N* = 120	
	Median		15	15	0.259 ^b^
	Mean		28	34	
	SD		36	40	
Waiting time at the facility (minutes)			*N* = 104	*N* = 120	
	Median		30	15	0.007 ^b^
	Mean		46	29	
	SD		53	39	
Satisfaction with the quality of health care received	Very good		11 (10.6%)	11 (9.2%)	
	Good		73 (70.2%)	74 (61.7%)	0.256 ^b^
	Normal		17 (16.3%)	34 (28.3%)	
	Poor		3 (2.9%)	1 (0.8%)	
Payment method for health services used			*N* = 104	*N* = 120	
	Social security scheme (SSS)	Yes = 1	–	109 (90.8%)	0.002 ^a^
		No = 0	104 (100%)	11 (9.2%)	
	Community-based health insurance (CBHI)	Yes = 1	–	–	
		No = 0	104 (100%)	120 (100%)	1.000 ^a^
	Out-of-pocket payments (OPPs)	Yes = 1	102 (98.1%)	110 (91.7%)	
		No = 0	2 (1.9%)	10 (8.3%)	0.034 ^a^
	Other (e.g. helping by relatives or employers)	Yes = 1	2 (1.9%)	–	
		No = 0	102 (98.1%)	120 (100%)	0.128 ^a^
Total out-of-pocket expenditure for health care received (MMK) ^c^ (includes respondents who used out-of-pocket payment to cover the expenditure for receiving health care)			*N* = 100 missing = 2	*N* = 109 missing = 1	
		Median	8000	4500	0.050 ^b^
		Mean	247,582	31,728	
		SD	1,091,442	88,410	
Amount of money paid out of pocket for medicinal used (MMK)^c^			*N* = 93 missing = 9	*N* = 106 missing = 4	
		Median	15,000	3000	0.039 ^b^
		Mean	192,113	37,073	
		SD	709,785	117,252	
Borrowed money to cover the expenditure of health care used			*N* = 102	*N* = 110	
		Yes	14 (13.7%)	14 (12.7%)	0.873 ^a^
		No	87 (85.3%)	95 (86.4%)	
		Missing	1 (1%)	1 (0.9%)	
Amount of borrowed money to cover the expenditure of health care used (MMK)^c^ (includes respondents who borrowed money to pay for receiving health care)			*N* = 14	*N* = 13 missing = 1	
		Median	72,500	40,000	0.119 ^b^
		Mean	527,286	90,192	
		SD	964,538	145,704	
Sold properties to cover the expenditure of health care used			N = 102	N = 110	
		Yes	2 (2.0%)	4 (3.6%)	0.620 ^a^
		No	79 (77.5%)	105 (95.5%)	
		Missing	21 (20.6%)	1 (0.9%)	
Amount of money from the sold assets to cover the expenditure of health care used (MMK) ^c^ (includes respondents who sold properties to pay for receiving health care)			N = 2	N = 3 missing = 1	
		Median	112,500	70,000	0.692 ^b^
		Mean	112,500	78,333	
		SD	123,744	57,951	

^a^ Mann-Whitney U Test; ^b^ Independent samples t-test; ^c^ Exchange rate 1000 MMK = 0.81 USD (2015)

More than 90% of the SSS respondents who received health care outside of the SSS clinic paid out of pocket for the last service use. Yet, a significantly higher share of the general population sample who used health care, report OOPPs (98.1%) for their last services use. Payment for services through a CBHI, is not reported in both samples.

Among those who paid out of pocket, the mean total OOPPs expenditure for health care used during the last episode of illness is eight times higher in the general population sample, i.e. 247,582 MMK, than in the SSS sample, i.e. 31,728 MMK (1 USD = MMK). The mean expenditure for pharmaceuticals used for the last episode of illness is 192,113 MMK for the general population sample and 37,073 MMK for the SSS sample. There are statistically significant differences (*p* < 0.05) in the mean total expenditure and mean pharmaceutical spending between the two samples. The share of respondents who needed to borrow money to cover the health care expenditure among the general population sample is similar to that in the SSS sample, i.e. 13.7 and 12.7% of those who paid for the last health care use respectively. The mean amount of money borrowed to cover health care expenditure for the last service use in the general population, is five times higher (527,286 MMK) than that in the SSS sample (90,192 MMK). Similarly, the share of respondents who needed to sell assets to cover their health care expenditure for the last service use is similar across the samples, i.e., 2 and 3.6% respectively. The mean amount of money from the sold assets to cover health care expenditure in the general population sample is 112,500MMK and among the SSS sample, it is 78,333 MMK.

### Results of the cluster analysis and regression analysis

**Fig. 1:**
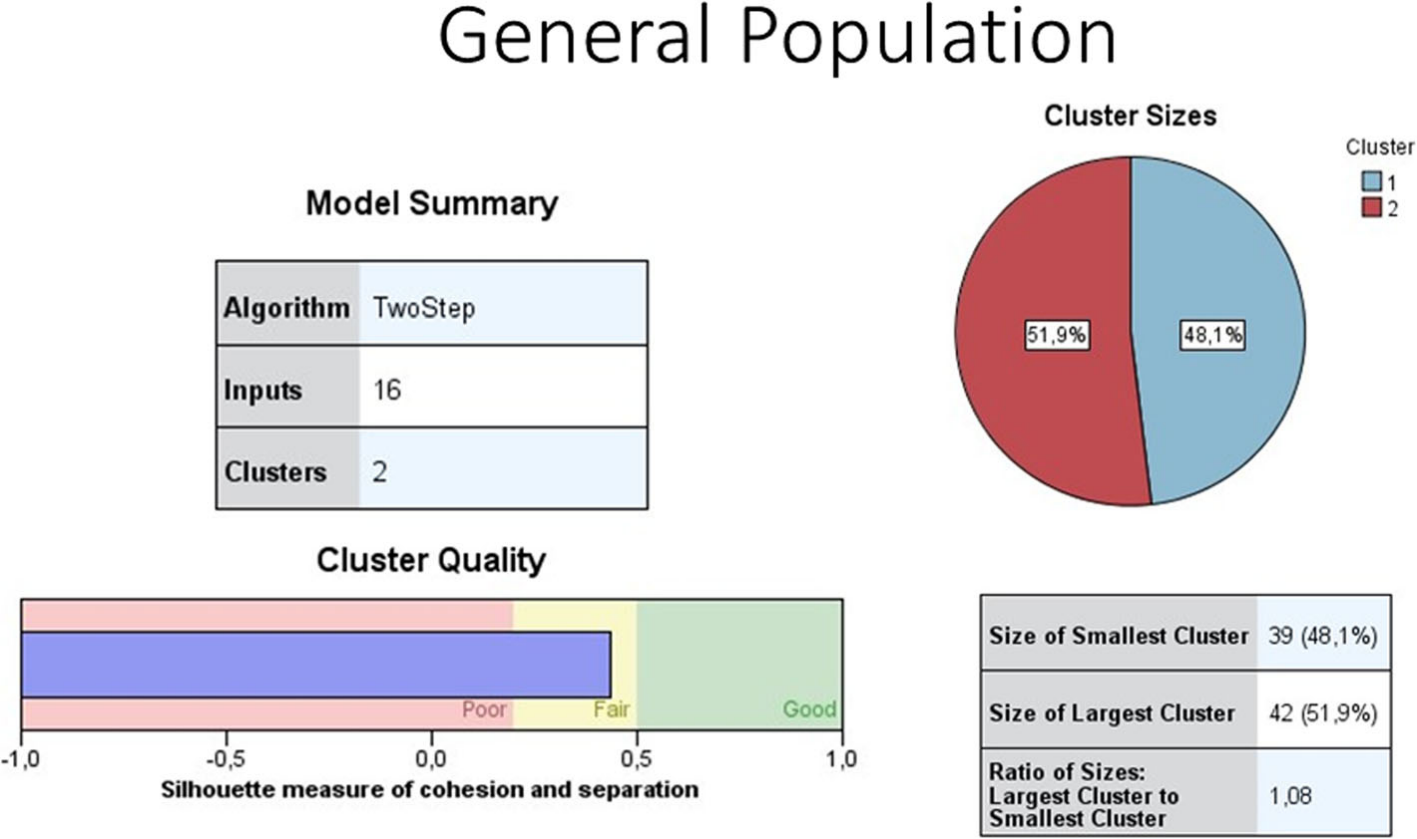
Model summary and cluster quality of cluster analysis I for general population

**Fig. 2:**
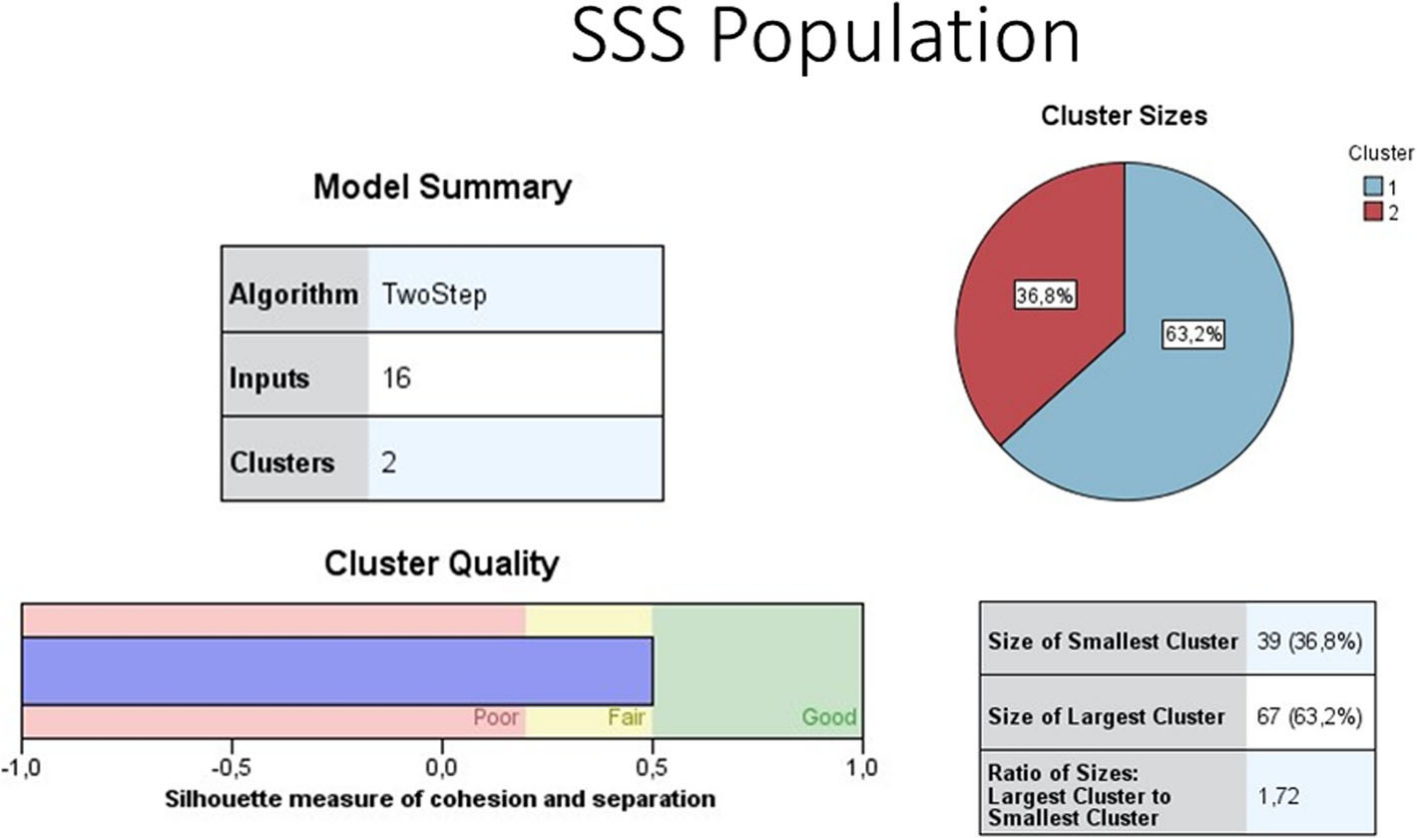
Model summary and cluster quality of cluster analysis I for SSS population

To identify patterns of health care utilization and payments, we performed four cluster analyses as described in the methods section. Table 4 presents the clusters generated by the two-step clustering procedure in each cluster analysis, including the size and main characteristics of the clusters. The clustering shows good quality (SI ≥ 0.5) for cluster analysis II, III, and IV, and fair quality (SI ≥ 0.2 and < 0.5) for cluster analysis I. The model summary and cluster quality of cluster analysis I for general population can be seen in Fig. 1 and that of SSS can be seen in Fig. 2. The model summary and cluster quality of cluster analysis II, III, and IV are described in Additional file 2. Detailed results of the four cluster analyses are shown in Additional file 3. Below we present the results of the cluster analysis I because this cluster analysis includes all variables.

**Table 4:** Results of the cluster analysis

Year of data collection 2015		Cluster composition describing dominant characteristics		Cluster size	
				*N*.	%
Cluster analysis I (all cluster variables: type of health care services used for last illness during past 12 months and reason of using such health care services; type of payment for health care services; and amount of payment and coping strategies)	General Sample	Cluster group 1	Last use of general care: Used health services at GP clinic or nearby health center or private specialist at outpatient department looked for closest facilities; out-of-pocket payments; required to borrow money to cover expenses	42	51.9
		Cluster group 2	Last use of specialized care/ hospitalized: Used private or public specialist services or hospitalization; looked for better quality of services or low cost, recommended or brought by someone; out-of-pocket payments; borrowed money or sold assets to cover high expenses	39	48.1
	SSS Sample	Cluster group 1	Last use of general care: Used health services at GP clinic or nearby health center; looked for closest facility or low cost; out-of-pocket payments only; required to borrow money to cover expenses	67	63.2
		Cluster group 2	Last use of specialized care/ hospitalized: Used health services at GP clinic or private/public specialist OPD or hospitalization; looked for better quality of service, use recommended or brought by someone, or other reasons; out-of-pocket payments; borrowed money or sold assets to cover high expenses	39	36.8
Cluster analysis II (type of health care services used for last illness during past 12 months and reason of using such health care services)	General Sample	Cluster group 1	Recommended public/private specialist care: Used private or public specialist service; recommended or brought by someone	15	14.4
		Cluster group 2:	Closest high quality health center: Used health services at a nearby health center or GP clinic; looked for the closest and quality service	23	22.1
		Cluster group 3	Nearby health center or GP or private specialist care: Used health services at nearby health center or GP clinic or private specialist OPD; because of other reasons	8	7.7
		Cluster group 4	High quality private specialist/ GP: Used health services at private specialist OPD or GP clinic; looked for the quality	22	21.2
		Cluster group 5	Low-cost health facility/ hospitalization: Used health services at GP clinic or nearby health center or hospitalization; looked for low cost	14	13.5
		Cluster group 6	Closest GP clinic: Used health services at GP clinic; looked for the closest facility	22	21.2
	SSS Sample	Cluster group 1	Closest facility: Used health services at GP clinic or nearby health center; looked for the closest facility	64	53.3
		Cluster group 2	Low cost, or quality service, or recommended or brought by someone;: Used health services at GP clinic, or public or private specialist OPD, or hospitalization; looked for the low cost, or quality service, or recommended or brought by someone	56	46.7
Cluster analysis III (type of payment for health care services)	General Sample	Cluster group 1	Out of Pocket Payments (OOPPs)	101	97.1
		Cluster group 2	Others (help from relatives or employers)	3	2.9
	SSS Sample	Cluster group 1	Out of Pocket Payments (OOPPs)	109	90.8
		Cluster group 2	SSS Payment only	11	9.2
Cluster analysis IV (amount of out-of-pocket payments and coping strategies)	General Population Sample	Cluster group 1	Low expense: Did not require to borrow money or sell assets to cover low expense	66	81.5
		Cluster group 2	High expense: Borrowed money or sold assets to cover high expense	15	18.5
	SSS Sample	Cluster group 1	Low expense: Did not require to borrow money or sell assets to cover low expense	84	79.2
		Cluster group 2	High expense: Borrowed money or sold assets to cover high expense	22	20.8

In cluster analysis I, for each sample, two distinct clusters are automatically generated based on the variables presented in Table 3. For both samples, cluster 1 includes respondents who report general care as the last use of care, and cluster 2 includes respondents who report specialized care or hospitalization as the last use of care. In the general population sample, the two clusters are of a fairly similar size (51.9 and 48.1% respectively). In the SSS sample, cluster 1 (general care), is nearly two times larger than cluster 2 (specialized care or hospitalization), 63.2 and 36.8% respectively. Generally, in both samples, respondents who belong to cluster 2 (specialized care or hospitalization), frequently indicate that the reason for using the services of the specific provider is because of their perception of good service quality or because the provider is recommended by someone. Those belonging to cluster 2 (specialized care or hospitalization) also often report that they needed to borrow money or sell assets to cover the high expenditure. In both samples, respondents who belong to cluster 1 (general care), explain the use of the specific provider mostly with its closeness to their homes. Respondents belonging to cluster 1 (general care) often make low OOPPs for health care and do not need to borrow or sell assets to pay for health care.

### Results of the binary logistic regression

As described in the method section, regression analyses were carried out to determine the association between the cluster membership and socio-demographic characteristics.

Table 5 presents the regression results related to cluster analysis I (the cluster analysis based on all variables in Table 4). For the general population sample, this regression analysis shows that cluster 1 (general care) includes more men while cluster 2 (specialist care and hospitalization) includes more women (OR = 3.269, *p* < 0.05). Also, cluster 1 (general care) includes more respondents whose household income does not allow savings while cluster 2 (specialist care and hospitalization) includes more respondents whose household income allows savings (OR = 4.120, *p* < 0.10). In the SSS sample, cluster 1 (general care) mostly consists of respondents with a middle or lower education level while cluster 2 (specialist care and hospitalization) mostly consists of respondents with a higher education level (OR = 2.282, *p* < 0.10). In addition, in the SSS sample, cluster 2 (specialist care and hospitalization) mostly consists of respondents whose household income is high (OR = 1.066 compared to respondents with higher education, *p* < 0.01).

**Table 5:** Binary logistic regression for cluster analysis I variables of both general and SSS samples

Independent variables	Cluster analysis I (all cluster variables: type of health care services used for last illness during past 12 months and reasons of utilization; type of payment for health care services; and amount of payment and coping strategies)	
	General Population Sample (*n* = 76) 1 = Cluster group 1 – Last use of general care 2 = Cluster group 2 – Last use of specialized care/hospitalized	SSS Sample (*n* = 106) 1 = Cluster group 1 – Last use of general care 2 = Cluster group 2 – Last use of specialized care/hospitalized
	Exp B (95% CI)	Exp B (95% CI)
Age	1.006(0.971–1.043)	0.966(0.912–1.023)
Gender (1-male; 2-female)	3.269(1.003–10.651)**	1.373(0.457–4.120)
What is your primary occupation activity at present? General population sample: 1-working; 2-not working^a^ SSS sample: 1-public; 2-private	0.643(0.212–1.948)	1.838(0.464–7.276)
What is your highest education level? 1-middle school and lower; 2-high school and higher^b^	1.082(0.356–3.292)	2.282(0.767–6.788)*
What is your civil status at present? 1-living alone; 2-living with spouse^c^	0.627(0.197–1.995)	0.983(0.306–3.157)
How would you rate your overall health status at present? 1-very poor and poor; 2-moderate; 3-good and very good	0.940(0.444–1.990)	0.707(0.361–1.387)
How many adult persons (age 18 or higher) are there in your household?	0.775(1.566–1.062)*	1.028(0.761–1.389)
How many children (under the age 18) are there in your household?	1.036(0.615–1.745)	0.784(0.505–1.218)
Considering the income of all household members and all sources of income (e.g. wages, social welfare, pensions, rents, fees, etc.), what is your average net monthly household income?	1.003(0.992–1.015)	1.066(1.025–1.110)***
Which of the following is true regarding your current household income? 1- does not allow to build savings; 2- allows to build savings^d^	4.120(0.776–21.876)*	1.448(0.514–4.080)
Constant	0.100	2.876
Nagelkerke R Square	0.182	0.213

*p < .10; **p < .05; ***p < .01

^a^ The working includes public, private, self-employed, family business, and others; the not working group includes pensioners, students, and unemployed

^b^ The group with middle school and lower includes Illiterate, primary school, and middle school; the group with high school and higher including high school, graduate and higher degree

^c^ The group living alone includes single, separated, divorced, widow; the group living with spouse including married and living with a partner without marriage

^d^ The group not able to build savings includes the following categories: just meets the expenses, not sufficient/ need to use savings, need to borrow

The regression results of cluster analysis II, III, and IV are presented in Additional file 4.

## Discussion

In this study, we have explored the patterns of health care utilization and payments during the last episode of illness among two samples — a general population sample who are not SSS members and a sample of SSS members. Respondents in the SSS sample, who used health care in the past 12 months, report payments for their last health care use through the SSS but also OOPPs. For the general population sample, only OOPPs are reported as a type of payment for health care. This finding highlights the problem of widespread OOPPs which can result in impoverishment and financial catastrophe for those affected [22]. Importantly however, the SSS sample also report paying for health care out of pocket, which shows the limitations to attain financial risk protection by the SSS. This is because SSS members utilize health care outside of SSS. This finding shows that the SSS needs to explore the causes of the low utilization rate of health care provided by the SSS and how much its members understand the reimbursement policy and the challenges in the process of reimbursement. At the same time more study is required to explore whether SSS members who use health care outside of the SSS do not apply for reimbursement. One study on the social security scheme in Thailand found that if the beneficiaries have multiple medical insurance they might use the one with the most favorable services [20]. Inaccessibility, perceived poor quality of care, limited information on the insurance scheme such as location of health facilities that they eligible for and where payroll deductions can take place are found to be related factors that are associated with the poor utilization of health care under the provided scheme [20].

Yet, the OOPPs reported by the SSS members who took health care outside of SSS during the past 12 months, are slightly less frequent and lower than the OOPPs reported by the general population sample because SSS provides reimbursement for using health care at public facilities. Moreover, the mean total OOPPs expenditure for health care used and the mean OOPPs for pharmaceuticals are significantly higher in the general population sample than in the SSS sample. These findings might imply that in Myanmar, the SSS contributes to the financial risk protection of health care users to a certain extent even though the coverage and utilization rate is low. This finding is in line with the study by [4] in India, who found that the government-funded health insurance scheme, the Vajpayee Arogyashree Scheme (VAS), provides financial risk protection [4].

The finding of the preference of using the nearest facility is not only indicative of the financial barriers but also of the physical barriers to access health facilities. Access to health care is a fundamental objective of the UHC and a factor to increase enrolment in health insurance. More obviously among the SSS respondents is that they prefer to use health care at a nearby clinic than at a SSS clinic which might be far even if they can get free health care in the latter facility. In Thailand, the nationwide expansion of primary care center together with a committed health workforce has acted as a platform to achieve UHC by promoting access to health care. As the evidence shows, inaccessibility is one of the factors influencing the low utilization rate of health care under the scheme [19, 20]. The results of our cluster analysis show that the perception of high quality contributes to the pattern of health care use because more than 60% of the respondents give distance and quality of care as a reason of choosing the specific provider. This finding is in line with prior expectations because quality of care plays a crucial role in the health improvement [1, 6, 9, 16].

The association between income and utilization of general/specialist care indicates the need for further study to ensure access to necessary health care without financial barriers. Meanwhile, there is a need to develop a proper referral system including the private sector in Myanmar because there is no clear information on using specialized care with a proper referral. This can create inefficiencies in the health system by increasing the workload in specialist care facilities. A proper referral system could reduce the burden on specialist care facilities as well as lead to a delay in seeking health care. Among the general population sample, the households with higher income paid more for their last health care use but had to borrow money or sell their assets to cover the payment. This finding brings out the requirement of financial protection as the need to sell assets are an indicator of catastrophic health care cost which may lead to impoverishment of the affected household.

At the same time, the lower spending on health care among the low income group is because of the inability to pay for high costs of health care. Self-rated health status is associated with the amount of payment and coping strategies among the SSS sample. Apart from the described findings we have found that the other demographic factors have no significant association with the type of health care utilization, type and amount of payment, and coping strategies.

The study has some limitations, such as the small sample sizes that carried out in only three out of 15 regions, which limits the generalization of the findings. As there are no existing data we could not standardize the demographic factors such as age, income and nature of the job, between the two samples. Thus, we could not isolate the contribution of the difference in mean age, income or nature of the job on the difference between health care utilization and health expenditure of the two samples in our study. Also, the study collected data on health care use and payments related to the last illness within the past 12 months, which can be related to recall bias. We also need to acknowledge possible sample selection bias as we systematically excluded the SSS insured when drawing the general population sample. Moreover, the communities were selected based on feasibility as recommended by the authorities. This means that the sample selection was not fully random and we may therefore not necessarily generalize results to the country as a whole. Our study only shows the OOPPs of SSS members who took health care outside the SSS but does not report on the additional payments among SSS members who took health care provided by SSS. This study only provides a base for a new nation-wide study with a broader scope and larger samples, to study the effect of SSS membership. According to the cluster analysis results, most of the health care used during the last episode of illness, is at primary care level and further research should focus on this type of service utilization to differentiate between primary and specialist services.

## Conclusion

The significantly higher OOPPs among general population sample highlighted the need of financial protection among this group. In addition, households with higher income have to pay higher expense for their last health care used and had to borrow money or sell assets as coping strategies to cover the payment is evidence of the high burden of the health care cost among Myanmar people. Low level of utilization of health care provided by social security scheme (23%) among its members who sought health care in the past 12 months shows the low preference of SSS service by its members. Myanmar requires extending social protection for both 1) coverage breadths as only 3.4% of the employment population enrolled in the SSS although we found that the SSS can provide financial risk protection to some extent, and 2) coverage depth as the SSS sample still need to find coping strategies to cover high OOPPs expenses for health care. Previous study, Tessier and Thida [21], also suggest that the SSS should try to extend the target groups who are easy to incorporate at first (i.e. families, civil servants) and then to other groups (i.e. smaller businesses, informal economy) in the coverage breaths aspect. The factors influencing choice of health services discovered by our study are distance, perceived high quality health care, and cost of health care in both samples. To strengthen SSS, the mentioned factors should be considered to improve.

## Additional files

Additional file 1: Survey questions. Description of data: 12 questions regards socio-demographic data and 14 questions regards utilization pattern of health care services with in past 12 months. (DOCX 47 kb)

Additional file 2: Model summary and cluster quality of cluster analysis II, III, IV. Description of data: Model summary and cluster quality of Cluster analysis II (Type of health services used for last illness during the past 12 months and reason of using these services) for both samples. Model summary and cluster quality of Cluster analysis III (Type of payment for healthcare services used) for both samples. Model summary and cluster quality of Cluster analysis IV (The amount of OOPPs and coping strategies). (DOCX 180 kb)

Additional file 3: Results of cluster analysis. Description of data: Results of cluster analysis I: all variables (General population and SSS population); Results of cluster analysis II (General population and SSS population): Type of health services used for last illness during past 12 months and reason of using such services (General population and SSS population); Results of cluster analysis III: Type of payment for healthcare services (General population and SSS population); Results of cluster analysis IV: Amount of payment and coping strategies (General population and SSS population). (DOCX 174 kb)

Additional file 4: Regression analysis for cluster group II, III, IV. Description of data: Multinomial logistic regression for cluster analysis II variables of general sample and binary logistic regression of SSS sample; Binary logistic regression for cluster analysis III and IV variables of both general and SSS samples; and Linear regression for amount of payment for medicines of both general and SSS samples. (DOCX 34 kb)

## Abbreviations

CBHI: Community-Based Health Insurance
GDP: Gross Domestic Product
GGE: General Government Expenditure
OOPPs: Out-of-pocket payments
SSS: Social Security Scheme
UHC: Universal Health Coverage
