# Supplementary material for: Patterns of health care use and out-of-pocket payments among general population and social security beneficiaries in Myanmar
Source: BMC Health Serv Res. 2019 Apr 27;19:258. doi: 10.1186/s12913-019-4071-8 (PMC6486983; doi:10.1186/s12913-019-4071-8)
Supplement: Supplementary file 1 — Survey questions. Description of data: 12 questions regards socio-demographic data and 14 questions regards utilization pattern of health care services with in past 12 months. (DOCX 47 kb) [file 12913_2019_4071_MOESM1_ESM.docx]

**SURVEY:** **PREFERENCES AND WILLINGNESS TO PAY FOR HEALTH INSURANCE IN MYANMAR**

**ROUND 1 / May-June 2015 / MYANMAR**

**FIELDWORK CARRIED OUT BY:**

**FIELDWORK MANAGED BY:**

| **The questionnaire should be filled in only for respondents who are 18 years old or older.** | | | | | | | | | | |
| --- | --- | --- | --- | --- | --- | --- | --- | --- | --- | --- |
|  | | | | | | | | | | |
|  | | | | | | | | | | |
| **Fill in the following information:** | | | | | | | | | | |
|  | | | | | | | | | | |
|  | | | | | | | | | | |
| **Sampling point** |  |  |  |  |  |  |  |  |  |  |
|  | | | | | | | | | | |
| **Region** |  |  |  |  |  |  |  |  |  |  |
|  | | | | | | | | | | |
| **Respondent ID number** |  |  |  |  |  |  |  |  |  |  |
|  | | | | | | | | | | |
| **Date of the interview  (dd/mm/yyyy)** |  |  | **/** |  |  | **/** | **2** | **0** | **1** | **5** |
|  | | | | | | | | | | |
| **Start time  (use 24 hours clock)** |  |  | **:** |  |  |  |  |  |  |  |
|  | | | | | | | | | | |
| **Interviewer ID number** |  |  |  |  |  |  |  |  |  |  |
| **Conduct the interview only if the respondent answers with YES in the Informed Consent Form.**  **Ask the questions following their order in the questionnaire.**  **Read the exact wording of the questions, and afterwards, if necessary, make clarifications.**  **Use local currency for all relevant questions (MMK).**  **Please try to avoid “don’t know” answers and refusals.**  **if the respondent refuses to answer, keep the answer box blank.**  **if the respondent responds with “don’t know”, fill in DNK in the box.** | | | | | | | | | | |
| © Maastricht University, 2015  No parts of this questionnaire may be used, translated, stored, published, copied or transmitted in any form and by any means (electronic, mechanical, copying, recording and etc.) without the written permission issued by .... | | | | | | | | | | |

**INFORMED CONSENT PROCEDURE**

- The aim of this survey is to collect data on the citizens’ opinion about the quality, access and price of medical services they use and about the characteristics of a future health insurance system in Myanmar.
- The survey is not commissioned by the government or a health insurer.
- This survey is part of an international research project at the Maastricht University, the Netherlands and funded by the NUFFIC. NUFFIC (the Dutch organization for internationalization of higher education) has awarded six Netherlands Fellowship Programme PhD grants to Maastricht University, four of which to the Faculty of Health, Medicine and Life Sciences. The Nuffic grant is a Dutch scholarship programme intended for mid-career professionals, originating from developing countries, who wish to carry out PhD research projects in the Netherlands.
- The data collected during the survey will be used for research purposes only, namely for statistical analyses and reports.
- Your answers will not be related to your personal details (address, etc.) and will be completely confidential.
- Answers to all questions are highly important to the project, so we hope that you will share your opinions and thoughts by answering all questions in the questionnaire.

**Informed consent:**

**0.1 Do you agree to participate in this survey?**

0.1.1 Yes
0.1.2 No

**0.2 Are you over 18 years old?**

0.2.1 Yes
0.2.2 No

**Section 1. Socio-demographic**

Finally, there are some questions about your socio-demographic characteristics. Please note that your data will be kept confidential. There are many individuals with the same socio-demographic characteristics. The complete information about socio-demographic characteristics will be highly important for our further analysis of the data.

**1. In which year were you born?**

1.1.................................. yyyy

**2. What is your gender?**

2.1 Male

2.2 Female

**3. What is the name of the Ward and or Township you live in?**

3.1..................................

**4. What is your primary occupation activity at present?**

4.1 Farmer
4.2 Soldier
4.3 Factory
4.4 self-employed
4.5 Family business
4.6 Public sector (government)

4.7 Private-cooperation

4.8 Student

4.9 Unemployed and looking for a job
4.10 Unemployed not looking for a job (e.g. housewife)

4.11 Pension (because of age)
4.12 Pension (because of illness)
4.13 other, specify ...................................

**5.What is your highest educational level?**

5.1 I never went to school

5.2 I never went to school, but I can read and write
5.3 primary school
5.4 middle school
5.5 high school
5.6 vocational training
5.7 graduate and higher degree (university degree)
5.8 other, specify ...................................

**6. What is your civil status at present?**

6.1 Never married and single

6.2 Living with a partner without marriage
6.3 Married (living together)
6.4 Married (living separately)
6.5 Divorced and single at present
6.6 Widow/er and single at present

**7. How would you rate your overall health status at present?**

7.1 Very poor

7.2 Poor

7.3 Moderate

7.4 Good

7.5 Very good

**8. Do you have any kind of social protection status?**

8.1 Formal sector

8.2 Informal sector

8.3 Civil servant

8.4 Other, please specify: …………………….

**9. How many adult persons (age 18 or higher) are there in your household?**

9.1 …………………….. adult household members

**10. How many children (under the age 18) are there in your household?**

10.1 …………………….. children in the household

**11. Considering the income of all household members and all sources of income (e.g. wages, social welfare, pensions, rents, fees, etc.), what is your average net monthly household income?**

11.1 ………………………. MMK

**12. Which of the following is true regarding your current household income?**

12.1 Allows to build savings

12.2 Allows to save just a little

12.3 Only just meets the expenses

12.4 Not sufficient / need to use savings

12.5 Not really sufficient / need to borrow

**Section 2. Past Behaviour**

The first set of questions concerns medical services that YOU used during the last 12 months

(June 2014 – May 2015), and the money that you paid for receiving these services.

1. **Did you experience any symptoms of illness during the past 12 months?**

1.1 Yes

1.2 No

If not, please go to section 3.

**2. Did you seek/receive healthcare services during the past 12 months (physician visit or hospitalization)?**

2.1 Yes

2.2 No, because I did not find it necessary

2.3 No, because I purchase medication myself

2.4 No, because of long distance

2.5 No, because of high out-of-pocket expenditures (translation, too much money)

- 1. No, due to another reason, please specify: ……………….

🡪If not, please go to section 3.

The following questions concern your last use of healthcare services.

**3. Which of the following service types can best describe your last use of healthcare services? (select only one service type related to the very last use of healthcare services)**

3.1 Visit to nearby health centre

3.2 Visit to general practitioner

3.3 Visit to outpatient medical specialist at public hospital

3.4 Visit to outpatient medical specialist at private hospital

3.5 Hospitalization (incl. one day hospitalization)

3.6 Traditional healer

3.7Other, please specify: ……………….

🡪If traditional healer or other, please go to section 3.

**4. Why did you choose this kind of health service?**

4.1 it was the closest facility

4.2 I had to pay less than in other facilities

4.3 I had to wait less than in other facilities

4.4 It provided the best quality services

4.5 It was recommended to me

4.6 I was brought there

4.7 Other, please specify: ……………….

**5. How far was this facility from your home in terms of travel/transportation time?**

5.1 __:__ HH:MM

**6. How long did you wait at the facility to meet with a healthcare provider?**

6.1 ………………. HH:MM

**7. Are you overall satisfied with the quality of healthcare received during your last use of healthcare services?**

7.1 Very good

7.2 Good

7.3 Normal

7.4 Poor

7.5 Very poor

**8. Why?**

8.1 ………………......................

**9. Which kind of methods were used to cover all costs related to your last use of healthcare services? (multiple answers possible)**

9.1 Social security scheme only (SSS)

9.2 Community based health insurance only (CBHI)

9.3 Out-of-pocket expenditures only (OOP)

9.4 Other, please specify: ……………………

🡪 If no OPP, please go to section 3.

The following questions concern the money that you paid for your last use of healthcare services.

**10. How much did you spend in total** **for your last use of healthcare services?**

10.1 ………………………. MMK

**11. How much of this was for pharmaceuticals (medicines)?**

11.1 ………………………. MMK

**12: Did you have to borrow money to cover the above expenses** **for your last use of healthcare services and pharmaceuticals?**

12.1 Yes

12.2 No

🡪 If yes:

**12A: From whom did you borrow?**

12A.1 ……………………….

**12B: How much of the borrowed money did you use to pay for your last use of healthcare services?**

12B.1 ………………………. MMK

**13: Did you have to sell assets to cover the above expenses for your last use of healthcare services?**

13.1 Yes

13.2 No

🡪 If yes:

**13A: What kind of assets did you sell?**

13A.1 ……………………….

**13B: How much of the money from the sold assets did you use to pay for your last use of healthcare services?**

13B.1 ………………………. MMK

**14: Did you use any other ways to cover the expenses for this last healthcare service?**

14.1 Yes, please specify: .....................................

14.2 No
