# Supplementary material for: Patterns of health care use and out-of-pocket payments among general population and social security beneficiaries in Myanmar
Source: BMC Health Serv Res. 2019 Apr 27;19:258. doi: 10.1186/s12913-019-4071-8 (PMC6486983; doi:10.1186/s12913-019-4071-8)
Supplement: Supplementary file 2 — Model summary and cluster quality of cluster analysis II, III, IV. Description of data: Model summary and cluster quality of Cluster analysis II (Type of health services used for last illness during the past 12 months and reason of using these services) for both samples. Model summary and cluster quality of Cluster analysis III (Type of payment for healthcare services used) for both samples. Model summary and cluster quality of Cluster analysis IV (The amount of OOPPs and coping strategies). (DOCX 180 kb) [file 12913_2019_4071_MOESM2_ESM.docx]

**Model summary and cluster quality of membership variables**

| General population | |
| --- | --- |
| 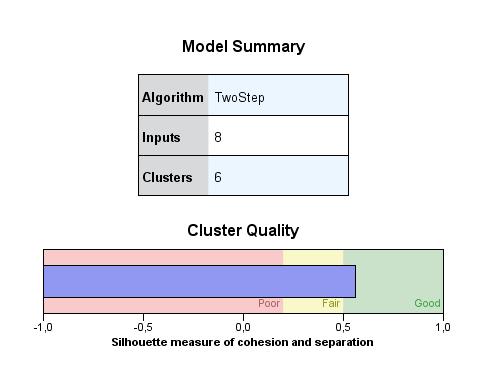 | 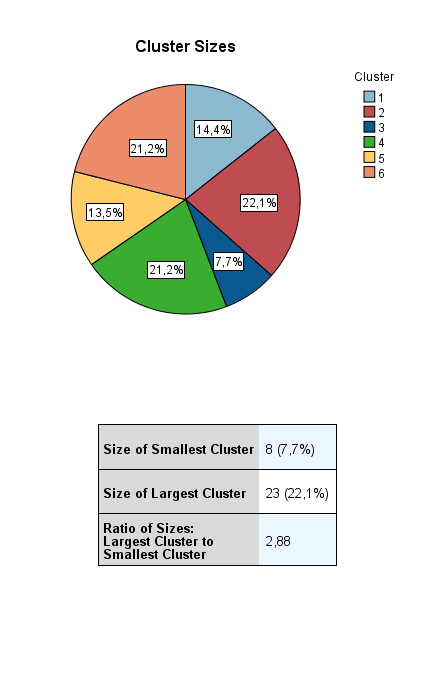 |
| SSS population | |
| 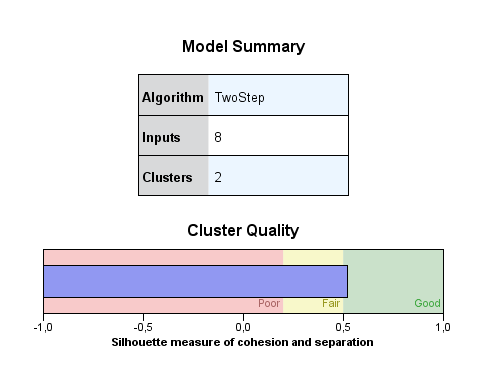 | 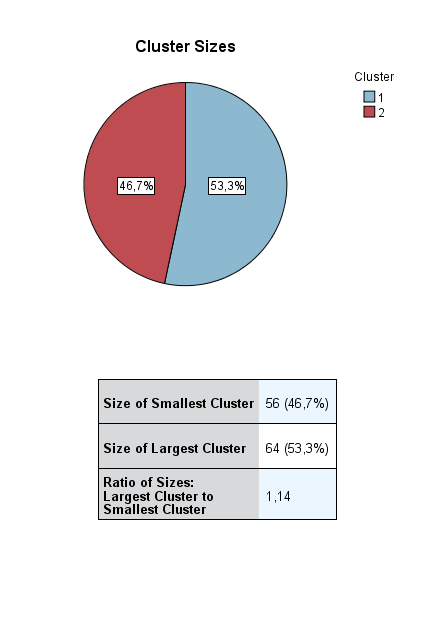 |

Cluster analysis II (Type of health services used for last illness during the past 12 months and reason of using these services)

Cluster analysis III (Type of payment for healthcare services used)

| General population | |
| --- | --- |
| 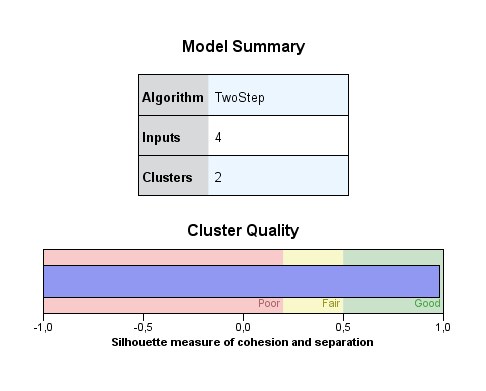 | 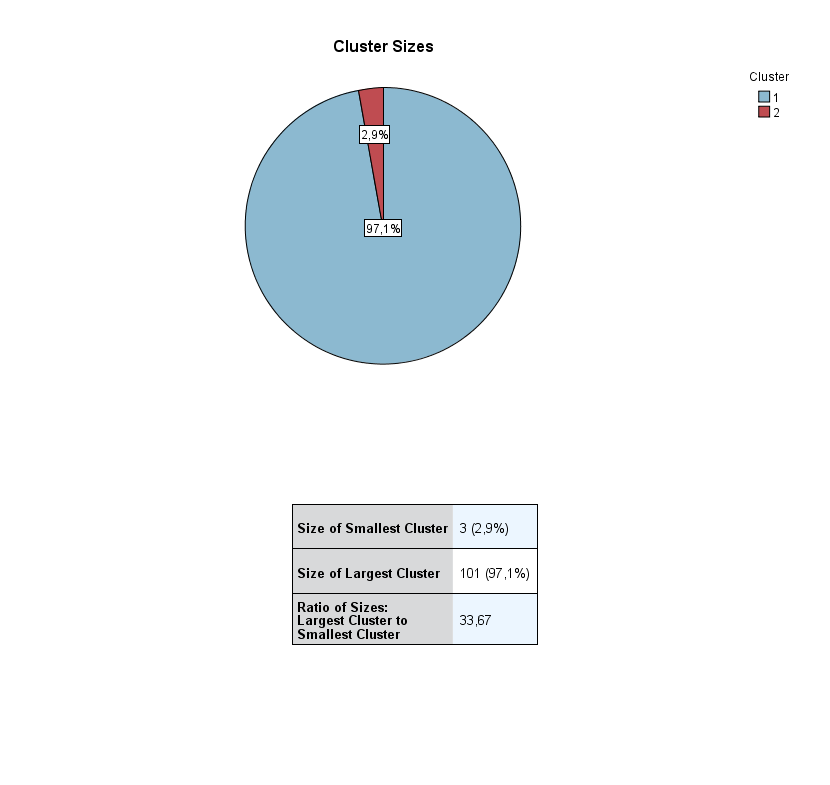 |
| SSS population | |
| 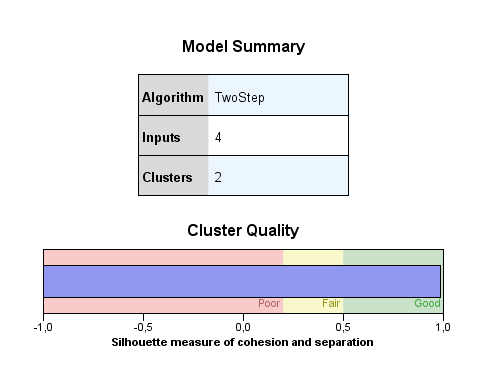 | 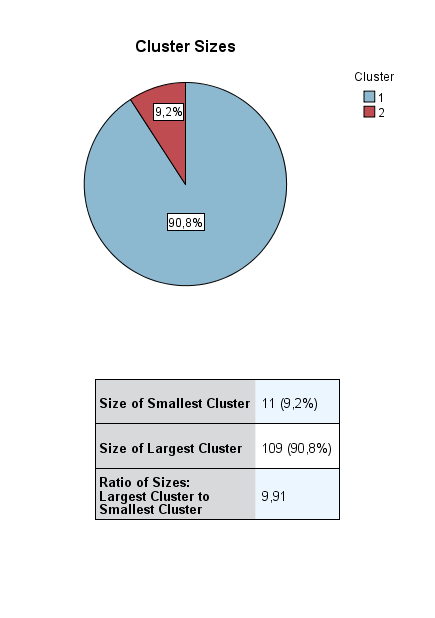 |

Cluster analysis IV (The amount of OOPPs and coping strategies)

| General population | |
| --- | --- |
| 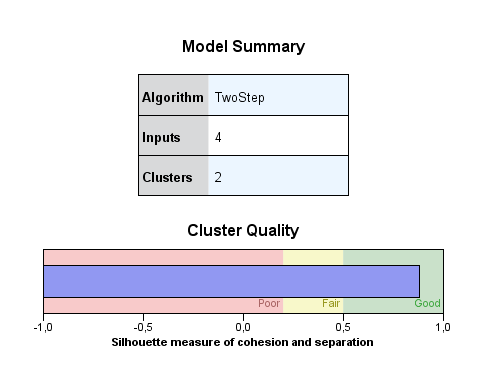 | 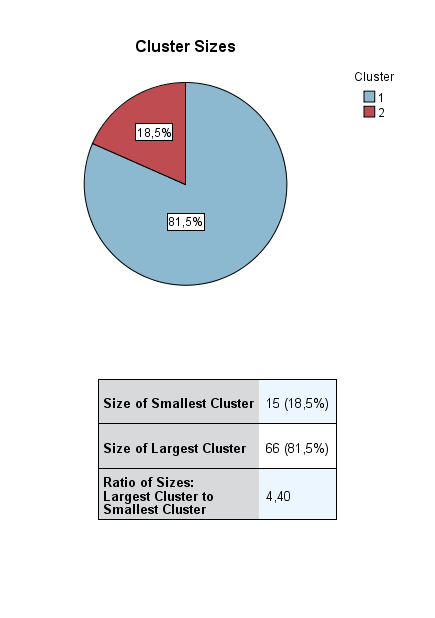 |
| SSS population | |
| 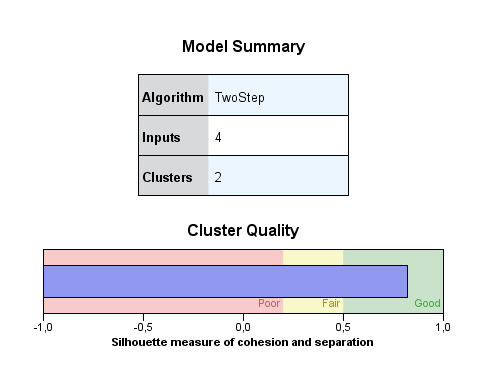 | 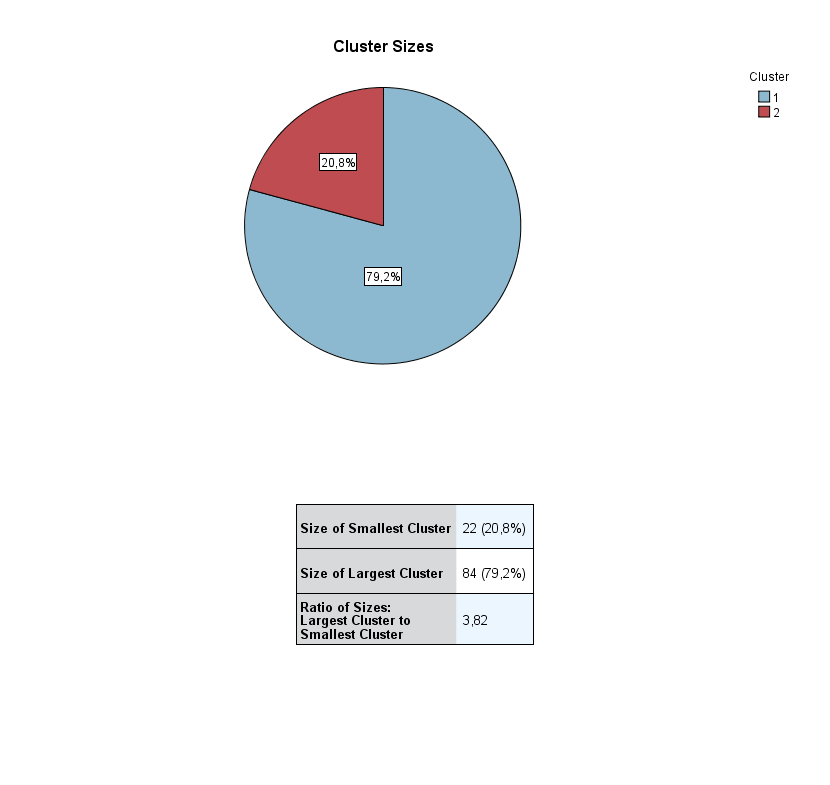 |
