# Supplementary material for: Patterns of health care use and out-of-pocket payments among general population and social security beneficiaries in Myanmar
Source: BMC Health Serv Res. 2019 Apr 27;19:258. doi: 10.1186/s12913-019-4071-8 (PMC6486983; doi:10.1186/s12913-019-4071-8)
Supplement: Supplementary file 3 — Results of cluster analysis. Description of data: Results of cluster analysis I: all variables (General population and SSS population); Results of cluster analysis II (General population and SSS population): Type of health services used for last illness during past 12 months and reason of using such services (General population and SSS population); Results of cluster analysis III: Type of payment for healthcare services (General population and SSS population); Results of cluster analysis IV: Amount of payment and coping strategies (General population and SSS population). (DOCX 174 kb) [file 12913_2019_4071_MOESM3_ESM.docx]

|  |  | General population | | SSS population | |
| --- | --- | --- | --- | --- | --- |
|  |  | Cluster group 1  N=39  n(%) | Cluster group 2  N=42  n(%) | Cluster group 1  N=67  n(%) | Cluster group 2  N=39  n(%) |
| Which of the following service types can best describe your last use of healthcare services? (select only one service type related to the very last use of healthcare services) | | | | | |
| Visit to nearby health center (HC) |  | 5(26.3%) | 14(73.7%) | 22(88.0%) | 3(12.0%) |
| Visit to general practitioner (GP) |  | 6(25.0%) | 18(75.0%) | 44(64.7%) | 24(35.3%) |
| Visit to outpatient medical specialist at public hospital |  | 7(87.5%) | 1(12.5%) | 0(0.0%) | 3(100.0%) |
| Visit to outpatient medical specialist at private hospital |  | 17(65.4%) | 9(34.6%) | 0(0.0%) | 6(100.0%) |
| Hospitalization |  | 4(100.0%) | 0(0.0%) | 1(25.0%) | 3(75.0%) |
| Why did you choose this kind of health service? | | | | | |
| It was the closest facility. | Yes | 0(0.0%) | 42(100.0%) | 66(97.1%) | 2(2.9%) |
|  | No | 39(100.0%) | 0(0.0%) | 1(2.6%) | 37(97.4%) |
| I had to pay less than in other facilities. | Yes | 8(72.7%) | 3(27.3%) | 5(71.4%) | 2(28.6%) |
|  | No | 31(44.3%) | 39(55.7%) | 62(62.6%) | 37(37.4%) |
| I had to wait less than in other facilities. | Yes | 1(100.0%) | 0(0.0%) | 0 (0.0%) | 1(100.0%) |
|  | No | 38(47.5%) | 42(52.5%) | 67(63.8%) | 38(36.2%) |
| It provided the best quality services. | Yes | 19(79.2%) | 5(20.8%) | 2(14.3%) | 12(85.7%) |
|  | No | 20(35.1%) | 37(64.9%) | 65(70.7%) | 27(29.3%) |
| It was recommended to me. | Yes | 6(85.7%) | 1(14.3%) | 0(0.0%) | 11(100.0%) |
|  | No | 33(44.6%) | 41(55.4%) | 67(70.5%) | 28(29.5%) |
| I was brought there. | Yes | 5(100.0%) | 0(0.0%) | 0(0.0%) | 5(100.0%) |
|  | No | 34(44.7%) | 42(55.3%) | 67(66.3%) | 34(33.7%) |
| Other | Yes | 4(80.0%) | 1(20.0%) | 0(0.0%) | 11(100.0%) |
|  | No | 35(46.1%) | 41(53.9%) | 67(70.5%) | 28(29.5%) |
| Which kinds of methods were used to cover all costs related to your last use of healthcare services? (multiple answers possible) | | | | | |
| Social Security Service (SSS) | Yes | 0(0.0%) | 0(0.0%) | 0(0.0%) | 0(0.0%) |
|  | No | 39(48.1%) | 42(51.9%) | 67(63.2%) | 39(36.8%) |
| Community Based Health Insurance (CBHI) | Yes | 0(0.0%) | 0(0.0%) | 0(0.0%) | 0(0.0%) |
|  | No | 39(48.1%) | 42(51.9%) | 67(63.2%) | 39(36.8%) |
| Out of pocket payment (OOPP) | Yes | 37(46.8%) | 42(53.2%) | 67(63.2%) | 39(36.8%) |
|  | No | 2(100.0%) | 0(0.0%) | 0(0.0%) | 0(0.0%) |
| Others (e.g. helping by relatives or employers) | Yes | 1(50.0%) | 1(50.0%) | 0(0.0%) | 0(0.0%) |
|  | No | 38(48.1%) | 41(51.9%) | 67(63.2%) | 39(36.8%) |
| Amount of money spent for using health care service | | | | | |
| How much did you spend in total for your last use of health care service? | Mean | 537948.72 | 59047.62 | 6676.12 | 11161.67 |
|  | SD | 1700167.11 | 234950.33 | 76705.13 | 136984.81 |
| How much of this was for pharmaceuticals (medicines)? | Mean | 391794.87 | 32928.57 | 6029.85 | 90402.56 |
|  | SD | 1063313.19 | 90953.26 | 11199.78 | 182076.44 |
| Coping strategy | | | | | |
| Did you have to borrow money to cover the above expenses for your last use of health care services and pharmaceuticals? | Yes | 8(61.5%) | 5(38.5%) | 9(64.3%) | 5(35.7%) |
|  | No | 31(45.6%) | 37(54.4%) | 58(63.0%) | 34(37.0%) |
| Did you have to sell assets to cover the above expenses for your last use of health care services? | Yes | 2(100.0%) | 0(0.0%) | 1(25.0%) | 3(75.0%) |
|  | No | 37(46.8%) | 42(53.2%) | 66(64.7%) | 36(35.3%) |

Table S7: Results of cluster analysis I: all variables

|  |  | General population | | | | | |
| --- | --- | --- | --- | --- | --- | --- | --- |
|  |  | Cluster group 1  N=15  n(%) | Cluster group 2  N=23  n(%) | Cluster group 3  N=8  n(%) | Cluster group 4  N=22  n(%) | Cluster group 5  N=14  n(%) | Cluster group 6  N=22  n(%) |
| Which of the following service types can best describe your last use of healthcare services? (select only one service type related to the very last use of healthcare services) | | | | | | | |
| Visit to nearby health center (HC) |  | 0(0.0%) | 12(57.1%) | 3(14.3%) | 2(9.5%) | 4(19.0%) | 0(0.0%) |
| Visit to general practitioner (GP) |  | 1(2.8%) | 0(0.0%) | 3(8.3%) | 5(13.9%) | 5(13.9%) | 22(61.1%) |
| Visit to outpatient medical specialist at public hospital |  | 3(37.5%) | 1(12.5%) | 0(0.0%) | 2(25.0%) | 2(25.0%) | 0(0.0%) |
| Visit to outpatient medical specialist at private hospital |  | 10(29.4%) | 9(26.5%) | 2(5.9%) | 13(38.2%) | 0(0.0%) | 0(0.0%) |
| Hospitalization |  | 1(20.0%) | 1(20.0%) | 0(0.0%) | 0(0.0%) | 3(60.0%) | 0(0.0%) |
| Why did you choose this kind of health service? | | | | | | | |
| It was the closest facility. | Yes | 1(1.8%) | 23(41.8%) | 1(1.8%) | 0(0.0%) | 8(14.5%) | 22(40.0%) |
|  | No | 14(28.6%) | 0(0.0%) | 7(14.3%) | 22(44.9%) | 6(12.2%) | 0(0.0%) |
| I had to pay less than in other facilities. | Yes | 0(0.0%) | 0(0.0%) | 0(0.0%) | 2(12.5%) | 14(87.5%) | 0(0.0%) |
|  | No | 15(17.0%) | 23(26.1%) | 8(9.1%) | 20(22.7%) | 0(25.0%) | 22(25.0%) |
| I had to wait less than in other facilities. | Yes | 2(40.0%) | 0(0.0%) | 0(0.0%) | 1(20.0%) | 2(40.0%) | 0(0.0%) |
|  | No | 13(13.1%) | 23(23.2%) | 8(8.1%) | 21(21.2%) | 12(12.1%) | 22(22.2%) |
| It provided the best quality services. | Yes | 2(6.9%) | 4(13.8%) | 0(0.0%) | 22(75.9%) | 0(0.0%) | 1(3.4%) |
|  | No | 13(17.3%) | 19(25.3%) | 8(10.7%) | 0(0.0%) | 14(18.7%) | 21(28.0%) |
| It was recommended to me. | Yes | 9(100.0%) | 0(0.0%) | 0(0.0%) | 0(0.0%) | 0(0.0%) | 0(0.0%) |
|  | No | 6(6.3%) | 23(24.2%) | 8(8.4%) | 22(23.2%) | 14(14.7%) | 22(23.2%) |
| I was brought there. | Yes | 6(100.0%) | 0(0.0%) | 0(0.0%) | 0(0.0%) | 0(0.0%) | 0(0.0%) |
|  | No | 9(9.2%) | 23(23.5%) | 8(8.2%) | 22(22.4%) | 14(14.3%) | 22(22.4%) |
| Other | Yes | 0(0.0%) | 0(0.0%) | 8(100.0%) | 0(0.0%) | 0(0.0%) | 0(0.0%) |
|  | No | 15(15.6%) | 23(24.0%) | 0(0.0%) | 22(22.9%) | 14(14.6%) | 22(22.9%) |

Table S8: Results of cluster analysis II (General population): Type of health services used for last illness during past 12 months and reason of using such services

Table S9: Results of cluster analysis II (SSS population): Type of health services used for last illness during past 12 months and reason of using such services

|  |  | SSS population | |
| --- | --- | --- | --- |
|  |  | Cluster group 1  N=64  n(%) | Cluster group 2  N=56  n(%) |
| Which of the following service types can best describe your last use of healthcare services? (select only one service type related to the very last use of healthcare services) | | | |
| Visit to nearby health center (HC) |  | 21(80.8%) | 5(19.2%) |
| Visit to general practitioner (GP) |  | 43(59.7%) | 29(40.3%) |
| Visit to outpatient medical specialist at public hospital |  | 0(0.0%) | 8(100.0%) |
| Visit to outpatient medical specialist at private hospital |  | 0(0.0%) | 7(100.0%) |
| Hospitalization |  | 0(0.0%) | 7(100.0%) |
| Why did you choose this kind of health service? | | | |
| It was the closest facility. | Yes | 64(91.4%) | 6(8.6%) |
|  | No | 0(0.0%) | 50(100.0%) |
| I had to pay less than in other facilities. | Yes | 1(9.1%) | 10(90.9%) |
|  | No | 63(57.8%) | 46(42.2%) |
| I had to wait less than in other facilities. | Yes | 0(0.0%) | 1(100.0%) |
|  | No | 64(53.8%) | 55(46.2%) |
| It provided the best quality services. | Yes | 0(0.0%) | 15(100.0%) |
|  | No | 64(61.0%) | 41(39.0%) |
| It was recommended to me. | Yes | 0(0.0%) | 13(100.0%) |
|  | No | 64(59.8%) | 43(40.2%) |
| I was brought there. | Yes | 1(14.3%) | 6(85.7%) |
|  | No | 63(55.8%) | 50(44.2%) |
| Other | Yes | 0(0.0%) | 16(100.0%) |
|  | No | 64(61.5%) | 40(38.5%) |

Table S10: Results of cluster analysis III: Type of payment for healthcare services

|  |  | General population | | SSS population | |
| --- | --- | --- | --- | --- | --- |
|  |  | Cluster group 1  N=101  n(%) | Cluster group 2  N=3  n(%) | Cluster group 1  N=109  n(%) | Cluster group 2  N=11  n(%) |
| Which kinds of methods were used to cover all costs related to your last use of healthcare services? (multiple answers possible) | | | | | |
| Social Security Service (SSS) | Yes | 0(0.0%) | 0(0.0%) | 0(0.0%) | 11(100.0%) |
|  | No | 101(97.1%) | 3(2.9%) | 109(100.0%) | 0(0.0%) |
| Community Based Health Insurance (CBHI) | Yes | 0(0.0%) | 0(0.0%) | 0(0.0%) | 0(0.0%) |
|  | No | 101(97.1) | 3(2.9%) | 109(90.8%) | 11(9.2%) |
| Out of pocket payment (OOPP) | Yes | 101(99.0%) | 1(1.0%) | 109(99.1%) | 1(0.9%) |
|  | No | 0(0.0%) | 2(100.0%) | 0(0.0%) | 10(100.0%) |
| Others (e.g. helping by relatives or employers) | Yes | 0(0.0%) | 2(100.0%) | 0(0.0%) | 0(0.0%) |
|  | No | 101(99.0%) | 1(1.0%) | 109(90.8%) | 11(9.2%) |

Table S11: Results of cluster analysis IV: Amount of payment and coping strategies

|  |  | General population | | SSS population | |
| --- | --- | --- | --- | --- | --- |
|  |  | Cluster group 1  N=66  n(%) | Cluster group 2  N=15  n(%) | Cluster group 1  N=84  n(%) | Cluster group 2  N=22  n(%) |
| Amount of money spent for using health care service | | | | | |
| How much did you spend in total for your last use of health care service? | Mean | 71689.39 | 1248566.67 | 6920.24 | 129886.36 |
|  | SD | 182026.32 | 2641168.57 | 8358.29 | 165149.93 |
| How much of this was for pharmaceuticals (medicines)? | Mean | 57439.39 | 858133.33 | 6407.14 | 154159.09 |
|  | SD | 126725.92 | 1626300.72 | 12232.24 | 223662.02 |
| Coping strategies | | | | | |
| Did you have to borrow money to cover the above expenses for your last use of health care services and pharmaceuticals? | Yes | 0(0.0%) | 13(100.0%) | 0(0.0%) | 14(100.0%) |
|  | No | 66(97.1%) | 2(2.9%) | 84(91.3%) | 8(8.7%) |
| Did you have to sell assets to cover the above expenses for your last use of health care services? | Yes | 0(0.0%) | 2(100.0%) | 0(0.0%) | 4(100.0%) |
|  | No | 66(83.5%) | 13(16.5%) | 84(82.4%) | 18(17.6%) |
