# Supplementary material for: Patterns of health care use and out-of-pocket payments among general population and social security beneficiaries in Myanmar
Source: BMC Health Serv Res. 2019 Apr 27;19:258. doi: 10.1186/s12913-019-4071-8 (PMC6486983; doi:10.1186/s12913-019-4071-8)
Supplement: Supplementary file 4 — Regression analysis for cluster group II, III, IV. Description of data: Multinomial logistic regression for cluster analysis II variables of general sample and binary logistic regression of SSS sample; Binary logistic regression for cluster analysis III and IV variables of both general and SSS samples; and Linear regression for amount of payment for medicines of both general and SSS samples. (DOCX 34 kb) [file 12913_2019_4071_MOESM4_ESM.docx]

Table S5: Multinomial logistic regression for cluster analysis II variables of general sample and binary logistic regression of SSS sample

| Cluster analysis II  (type of health care services used for last illness during past 12 months and reasons of utilization) | | | | | | | | | | | |
| --- | --- | --- | --- | --- | --- | --- | --- | --- | --- | --- | --- |
| Variables | General Population Sample  (n=105)  Reference cluster: use GP because of closest facility | | | | | | | | |  | SSS Sample (n=120)  1 = Cluster group 1 –**closest facility**  2 = Cluster group 2 –. **Low cost, or quality service, or recommended or brought by someone** |
|  | **Recommended public/private specialist care** |  | **Closest high quality health center** |  | **Nearby health center or GP or private specialist care** |  | **High quality private specialist/ GP** |  | **Low-cost health facility/ hospitalization** |  | **Closest facility** |
|  | Exp B (95% CI) |  | Exp B  (95% CI) |  | Exp B  (95% CI) |  | Exp B  (95% CI) |  | Exp B  (95% CI) |  | Exp B  (95% CI) |
| Age | 1.052  (0.995-1.112)* |  | 1.047  (0.991-1.107)* |  | 1.050  (0.974-1.132) |  | 1.008  (0.955-1.064) |  | 1.053  (0.993-1.116)* |  | 1.000  (0.955-1.048) |
| Gender (1-male; 2-female) | 3.670  (0.571-23.592) |  | 1.893  (0.356-10.069) |  | 0.624  (0.58-6.662) |  | 4.218  (0.755-23.556)* |  | 4.155  (0.642-26.871)* |  | 1.293  (0.505-3.308) |
| What is your primary occupation activity at present?  General population sample: 1-working; 2-not working ^a^  SSS sample: 1-public; 2-private | 0.757  (0.138-4.140) |  | 0.716  (0.149-3.442) |  | 0.156  (0.015-1.656)* |  | 1.004  (0.213-4.728) |  | 0.408  (0.068-2.450) |  | 1.277  (0.403-4.053) |
| What is your highest education level?  1-middle school and lower; 2-high school and higher ^b^ | cannot be included  in the model |  | cannot be included  in the model |  | cannot be included  in the model |  | cannot be included  in the model |  | cannot be included  in the model |  | 2.182  (0.855-5.570)* |
| What is your civil status at present?  1-living alone; 2-living with spouse ^c^ | 1.970  (0.292-13.314) |  | 4.130  (0.681-25.065)* |  | 0.374  (0.021-6.759) |  | 0.925  (0.135-6.314) |  | 2.234  (0.318-15.678) |  | 1.199  (0.451-3.188) |
| How would you rate your overall health status at present?  1-very poor and poor; 2-moderate;  3-good and very good | 0.600  (0.187-1.926) |  | 0.604  (0.213-1.712) |  | 1.485  (0.359-6.139) |  | 0.434  (0.152-1.240)* |  | 0.839  (0.253-2.783) |  | 0.898  (0.507-1.589) |
| How many adult persons (age 18 or higher) are there in your household? | 0.845  (0.521-1.372) |  | 1.337  (0.868-2.059) |  | 0.818  (0.414-1.616) |  | 0.732  (0.456-1.174) |  | 1.034  (0.632-1.690) |  | 0.995  (0.762-1.299) |
| How many children (under the age 18) are there in your household? | 1.198  (0.581-2.471) |  | 0.621  (0.284-1.356) |  | 0.456  (0.141-1.472) |  | 0.785  (0.401-1.540) |  | 1.049  (0.469-2.345) |  | 1.033  (0.706-1.512) |
| Considering the income of all household members and all sources of income (e.g. wages, social welfare, pensions, rents, fees, etc.), what is your average net monthly household income? | 0.990  (0.958-1.024) |  | 1.009  (0.991-1.027) |  | 1.004  (0.980-1.029) |  | 1.014  (0.997-1.032)* |  | 0.973  (0.931-1.017) |  | 1.046  (1.011-1.082)*** |
| Which of the following is true regarding your current household income?  1- does not allow to build savings; 2- allows to build savings ^d^ | 1.958  (0.144-26.567) |  | 0.246  (0.034-1.761) |  | 1.925  (0.115-32.250) |  | 2.510  (0.333-18.932) |  | 1.456  (0.98-21.528) |  | 1.293  (0.538-3.106) |
| Constant  Nagelkerke (Pseudo R Square)  Nagelkerke R Square | |  |  |  |  |  |  |  | 0.479 |  | 1.107  0.117 |

p *<.10; **p< .05; ***p<.01

^a^ The working includes public, private, self-employed, family business, and others; the not working group includes pensioners, students, and unemployed

^b^ The group with middle school and lower includes Illiterate, primary school, and middle school; the group with high school and higher including high school, graduate and higher degree

^c^ The group living alone includes single, separated, divorced, widow; the group living with spouse including married and living with a partner without marriage

^d^ The group not able to build savings includes the following categories: just meets the expenses, not sufficient/ need to use savings, need to borrow

Table 6: Binary logistic regression for cluster analysis III and IV variables of both general and SSS samples

| Independent variables | Cluster analysis III  (type of payment for health care services) | | |  | Cluster analysis IV  (amount of payment and coping strategies) | | |
| --- | --- | --- | --- | --- | --- | --- | --- |
|  | General Population Sample  (n=95)  1 = Cluster group 1 – out-of-pocket payments  2= Cluster group 2 –Others payment mechanisms |  | SSS Sample  (n=120)  1 = Cluster group 1 – out-of-pocket payments  2= Cluster group 2 –contributions for SSS |  | General Population Sample  (n=76)  1= Cluster group 1- low expense  2= Cluster group 2-high expense |  | SSS Sample  (n=106)  1 = Cluster group 1- low expense  2= Cluster group 2-high expense |
|  | Exp B (95% CI) |  | Exp B (95% CI) |  | Exp B (95% CI) |  | Exp B (95% CI) |
| Age | 1.011(0.929-1.099) |  | 1.073(0.984-1.171) |  | 1.041(0.995-1.090)* |  | 1.008  (0.942-1.079) |
| Gender (1-male; 2-female) | 6.082(0.294-126.002) |  | 0.499(0.085-2.926) |  | 2.866(0.668-12.293) |  | 0.858  (0.202-3.641) |
| What is your primary occupation activity at present?  General population sample: 1-working; 2-not working ^a^  SSS sample: 1-public; 2-private | 2.337(0.105-51.854) |  | 2.035(0.339-12.205) |  | 0.751(0.166-3.395) |  | 3.493  (0.674-18.104)* |
| What is your highest education level?  1-middle school and lower; 2-high school and higher ^b^ | 1.403(0.092-21.384) |  | 0.709(0.128-3.936) |  | 0.802(0.205-3.142) |  | 3.124  (0.907-10.760)* |
| What is your civil status at present?  1-living alone; 2-living with spouse ^c^ | ^cannot be included in the model  due to lack of variations^ |  | 1.541(0.284-8.364) |  | ^cannot be included in the model  due to lack of variations^ |  | 0.931  (0.204-4.249) |
| How would you rate your overall health status at present?  1-very poor and poor; 2-moderate;  3-good and very good | 1.687(0.526-5.404) |  | 0.550(0.220-1.511) |  | 1.017(0.365-2.830) |  | 0.311  (0.136-0.713)*** |
| How many adult persons (age 18 or higher) are there in your household? | 1.096(0.523-2.298) |  | 0.703(0.382-1.295) |  | 1.087(0.706-1.673) |  | 1.042  (0.717-1.513) |
| How many children (under the age 18) are there in your household? | 1.687(0.526-5.404) |  | 1.571(0.785-3.144) |  | 1.175(0.590-2.342) |  | 1.110  (0.642-1.920) |
| Considering the income of all household members and all sources of income (e.g. wages, social welfare, pensions, rents, fees, etc.), what is your average net monthly household income? | 1.005(0.980-1.030) |  | 0.994(0.931-1.062) |  | 0.956(0.916-0.999)** |  | 0.970  (0.919-1.024) |
| Which of the following is true regarding your current household income?  1- does not allow to build savings; 2- allows to build savings ^d^ | ^cannot be included in the model  due to lack of variations^ |  | 0.494(0.107-2.288) |  | ^cannot be included in the model  due to lack of variations^ |  | 1.681  (0.420-6.724) |
| Constant  Nagelkerke R Square | 0.000  0.478 |  | 0.019  0.219 |  | 0.163  0.291 |  | 35.299  0.436 |

*p <.10; **p< .05; ***p<.01;

^a^ The working includes public, private, self-employed, family business, and others; the not working group includes pensioners, students, and unemployed

^b^ The group with middle school and lower includes Illiterate, primary school, and middle school; the group with high school and higher including high school, graduate and higher degree

^c^ The group living alone includes single, separated, divorced, widow; the group living with spouse including married and living with a partner without marriage

^d^ The group not able to build savings includes the following categories: just meets the expenses, not sufficient/ need to use savings, need to borrow

Table 7: Linear regression for amount of payment for medicines of both general and SSS samples

| Independent variables | General Population Sample  (n=76) | |  | SSS Sample  (n=106) | |
| --- | --- | --- | --- | --- | --- |
|  | Coef. | SE |  | Coef. | SE |
| Age | 7678.105 | 5833.638 |  | 124.135 | 1372.546 |
| Gender (1-male; 2-female) | -58697.421 | 192021.645 |  | -1458.715 | 26821.292 |
| What is your primary occupation activity at present?  General population sample: 1-working; 2-not working ^a^  SSS sample: 1-public; 2-private | 43859.806 | 181855.376 |  | 2069.620 | 34796.437 |
| What is your highest education level?  1-middle school and lower; 2-high school and higher ^b^ | 72394.949 | 183546.082 |  | -51143,254* | 27617.880 |
| What is your civil status at present? 1-living alone; 2-living with spouse ^c^ | -212527.038 | 196088.681 |  | 18026.059 | 28874.500 |
| How would you rate your overall health status at present? 1-very poor and poor; 2-moderate; 3-good and very good | 14848.391 | 122211.833 |  | -44068.415** | 16705.662 |
| How many adult persons (age 18 or higher) are there in your household? | -847.470 | 48806.291 |  | -6940.981 | 7835.397 |
| How many children (under the age 18) are there in your household? | -40565.305 | 87049.097 |  | -19166.944 | 10946.103 |
| Considering the income of all household members and all sources of income (e.g. wages, social welfare, pensions, rents, fees, etc.), what is your average net monthly household income? | 1037.412 | 1868.582 |  | 1453.305 | 907.214 |
| Which of the following is true regarding your current household income?  1- does not allow to build savings; 2- allows to build savings ^d^ | -145901.802 | 250148.672 |  | -16204.701 | 25836.653 |
| Constant  Prob> F 0.000  R-squared 0.203  Adj R-Squared 0.156 | 232168.751  0.532  0.065  -0.057 | 877836.362 |  | 211675.101  F 1.979 **  0.172  0.085 | 143597.222 |

*p <.10; **p< .05; ***p<.01

^a^ The working includes public, private, self-employed, family business, and others; the not working group includes pensioners, students, and unemployed

^b^ The group with middle school and lower includes Illiterate, primary school, and middle school; the group with high school and higher including high school, graduate and higher degree

^c^ The group living alone includes single, separated, divorced, widow; the group living with spouse including married and living with a partner without marriage

^d^ The group not able to build savings includes the following categories: just meets the expenses, not sufficient/ need to use savings, need to borro
